# Medical Colleges and Quackery

**Published:** 1882-07

**Authors:** 


### MEDICAL COLLEGES AND QUACKERY.

The ostensible object of medical colleges is the education of men in the science and art of medicine in its widest and most comprehensive sense. It is presumed that the young men who place themselves under the pupilage of these institutions will be carefully nurtured and trained in all the intricacies of a difficult and inexact science; that correct principles will be inculcated; that a becoming reverence for the profession into which they are about to enter will be instilled, and that a decorous deportment under the varying and often trying scenes through which they soon must be called to pass, will be counseled.

All this, and much more, is understood to be taught the faithful student in lectures didactic and lectures clinical, both by precept and by example, so that when the formal degree, for which he has honestly toiled, is conferred, he may leave his alma mater imbued with the honorable character of his calling, stimulated by high aspirations, strengthened by noble examples and surrounded by true and helpful associates—men, who like himself, have espoused a worthy cause and have entered the ranks of a learned profession, honorable and honored, to engage in commendable rivalry with each other, and, emulous of preferment, to strive for eminence and success, which thus attained means merit—merit recognized and rewarded.

Having once felt these impulses and being still impressed with the value of such influences, our astonishment can be imagined when, only a few days ago, we discovered in the list of recent graduates of an old and well-known medical college the name of a notorious nostrum vendor. Standing side by side with aspiring and worthy young men, he received the indorsement of a corps of dignified professors and was sent out to the world with the seal of the institution upon him, not to practice medicine, ah, no; not that, no such drudgery for him; but, under cover of a diploma, unconditionally granted, to continue to ply his vocation undisturbed, and to continue to dispense his secret compound without the fear of the law before his eyes.

We are not apprised of the arguments advanced by these gentlemen, if any there were, to explain or excuse this act. We are merely dealing with the fact as it appears in their printed announcement and with the principle which the act involves, and whatever motive may have prompted them to lend their aid to the accomplishment of this unhallowed end, we emphatically assert that it was not considerate of the class; it was not complimentary to the alumni; it can scarcely reflect credit upon the college, and it surely will not tend to exalt the standing of the medical profession.

We insist that the same obligation to uphold and promote the dignity and usefulness of the profession rests upon the professors in medical colleges, which attaches to other medical men. Medical faculties are not, by virtue of their office, absolved from this unmistakable duty or from their allegiance to the medical profession, and they are not under any conceivable circumstances, justified in legalizing disreputable practices, licensing premeditated quackery, or contravening a salutary legal enactment.

Possibly the members of this faculty esteem their diplomas so lightly that they consider them merely a certificate of attendance upon two courses of lectures. But the law contemplates something more than this, when it makes a medical diploma, lawfully conferred, a license to practice medicine in this state. And just here, the question naturally arises, if this is the view entertained by any college, does its diploma, under the law, entitle the holder to the privileges and immunities of a legally qualified physician?

